# Hyperoxemia – too much of a good thing?

**DOI:** 10.1186/s13054-014-0556-3

**Published:** 2014-10-07

**Authors:** Hayley Gershengorn

**Affiliations:** Division of Critical Care Medicine and Department of Neurology, Albert Einstein College of Medicine, Montefiore Medical Center, 111 East 210th Street, Gold Zone, Main Floor, Bronx, NY 10467 USA

## Abstract

While avoiding hypoxemia has long been a goal of critical care practitioners, less attention has been paid to the potential for excessive oxygenation. Interest has mounted recently in understanding the clinical effects of hyperoxemia during critical illness, in particular its impact following cardiac arrest. In this issue of *Critical Care*, Dell’Anna and colleagues review available animal and human data evaluating the impact of hyperoxemia after cardiac arrest. They conclude that while hyperoxemia during cardiopulmonary resuscitation is probably desirable, it should probably be avoided during post-resuscitation care. These conclusions are in line with two broader themes in contemporary critical care: that less may be more; and that it is time to look beyond simply preventing short-term mortality towards longer-term outcomes.

Interest has mounted recently in understanding the clinical effects of hyperoxemia during critical illness, particularly its impact following cardiac arrest. In this issue of *Critical Care*, Dell’Anna and colleagues review available animal and human data evaluating the impact of hyperoxemia after cardiac arrest [1].

When patients are acutely critically ill, initial interventions must be directed towards immediately life-threatening issues. Significant hypoxemia can quickly lead to cardiac arrest, so early aggressive supplemental oxygen is frequently provided either in response to or for prevention of dangerous reductions in the arterial partial pressure of oxygen. Once a patient is stabilized and the focus appropriately turns to urgent diagnosis and treatment, however, often little effort is made to minimize the amount of supplemental oxygen delivered. In fact, the majority of mechanically ventilated patients continue to receive excess supplemental oxygen throughout their ICU stay [2,3]. Adverse effects of excess oxygen are best understood in the brain and the systemic circulation. Hyperoxemia can induce cerebral vasoconstriction [4], neuronal cell death [5], and seizures [6,7]. In addition, hyperoxemia reduces the cardiac index and heart rate while increasing peripheral vascular resistance [8,9]. Given the major neurologic and hemodynamic challenges faced by many critically ill patients, hyperoxemia may be especially concerning in this population.

In their article, Dell’Anna and colleagues provide an excellent perspective on hyperoxemia following cardiac arrest [1]. After exploring the pathophysiology of hyperoxemia in the setting of ischemia–reperfusion brain injury, they detail animal and human studies of hyperoxemia following cardiac arrest. They conclude that while hyperoxemia is probably prudent during resuscitation, avoiding hyperoxemia is probably desirable in the post-resuscitation phase. Most importantly, however, they highlight the limits of our current knowledge – for example, is there a safe upper limit for arterial partial pressure of oxygen? Is even a single episode of hyperoxemia detrimental? What is the role of carbon dioxide? – and wisely call for further study. Of note, a recent meta-analysis based on many of the same studies reviewed by Dell’Anna and colleagues found that while hyperoxemia following cardiac arrest was associated with an increased risk of in-hospital mortality (odds ratio: 1.40, 95% confidence interval: 1.02 to 1.93), the heterogeneity among studies precluded firm conclusions about the practice [10].

In focusing on the potential downsides of post-arrest hyperoxemia, Dell’Anna and colleagues hit on two important themes of current critical care research and practice. First, they implore us to consider the idea that doing more is not necessarily in our patients’ best interest – a concept that has taken hold recently in the critical care community. In the United States, the Critical Care Societies Collaborative’s contribution to the Choosing Wisely Campaign suggests consideration of the merits of doing less [11]. For example, influential studies have suggested benefits associated with less aggressive transfusion practices [12] or use of less sedation during mechanical ventilation [13]. While blood transfusion and sedation are clearly needed for some cases of anemia and agitation, respectively, too much of either can cause harm. Critical care has evolved to include doing less, in many cases, as a thoughtful alternative to doing more – not only when goals of care are palliation, but also when the goal is to increase chances of survival or to improve other clinical outcomes.

A second theme addressed by this perspective is the focus on long-term goals versus short-term goals. In evaluating the impact of hyperoxemia after initial resuscitation, Dell’Anna and colleagues shift focus beyond return of spontaneous circulation to include the impact on post-arrest outcomes. More broadly, this shift in focus can be seen in the critical care community as interest moves from solely evaluating short-term (in-hospital or 30-day) survival to longer-term survival (months rather than weeks or days) and alternative patient-centered outcomes such as quality of life and functional recovery [14,15]. Having improved our ability to achieve the traditional primary mission in critical care – keeping people alive– we now turn our knowledge, insights and attention to optimizing what it means to be a survivor.

Hyperoxemia in the post-resuscitation phase following cardiac arrest is probably detrimental, yet the nuances of this association are as yet unknown. As Dell’Anna and colleagues state, further study is certainly needed to fine-tune our understanding. With such insight we will hopefully learn at what point ‘enough’ oxygen becomes ‘too much’ and what impact ‘too much’ has on short-term survival, long-term survival, and quality of life.
